# Neurogenic Bladder Repair Using Autologous Mesenchymal Stem Cells

**DOI:** 10.1155/2016/2539320

**Published:** 2016-08-30

**Authors:** Pradeep V. Mahajan, Swetha Subramanian, Amit Danke, Anand Kumar

**Affiliations:** StemRx Bioscience Solutions Pvt. Ltd., Navi Mumbai, India

## Abstract

The normal function of the urinary bladder is to store and expel urine in a coordinated, controlled fashion, the activity of which is regulated by the central and peripheral nervous systems. Neurogenic bladder is a term applied to a malfunctioning urinary bladder due to neurologic dysfunction or insult emanating from internal or external trauma, disease, or injury. This report describes a case of neurogenic bladder following laminectomy procedure and long-standing diabetes mellitus with neuropathy treated with autologous cellular therapy. The differentiation potential and paracrine effects of mesenchymal stem cells on bladder function have been highlighted.

## 1. Introduction

Lumbar spinal stenosis is a condition, common among elderly persons, which causes intractable leg and back pain. Decompressive laminectomy is the procedure advised that creates space in the spinal canal to relieve pressure in the spinal cord. However, the procedure is associated with a high incidence of neurogenic dysfunction of the detrusor muscle (hypo- or hypercontractility), which is the muscle associated with voiding of urine [1]. Apart from impaired bladder compliance, detrusor dysfunction of prolonged duration may lead to hydronephrosis and renal deterioration.

The present goals of the management of neurogenic bladder are the preservation of the renal function and increasing the quality of life for patients by minimizing complications. However, it is not possible to achieve bladder function restoration and tissue repair as these techniques do not result in neuronal regeneration.

Mesenchymal stem cells (MSCs) augment healing through cell replacement and stimulation of cell proliferation and angiogenesis. We hereby present a case of neurogenic bladder successfully managed with autologous mesenchymal stem cells.

## 2. Case Report

A 65-year-old male patient had complaints of inability to micturate since 3 years, despite presence of sensation of bladder fullness. He has diabetes mellitus and hypertension since 14 years. Past history revealed that the patient had complaints of pain and numbness/tingling sensation in his lower back and limbs before 3-4 years. Following investigations, it was revealed that the patient had disc herniation in his lumbar region and laminectomy procedure was recommended to him. This procedure as well as long-standing uncontrolled diabetes and neuropathy led to gradual loss of bowel and bladder function. His ability to micturate voluntarily was lost completely within a year following laminectomy procedure and he would frequently get constipated. Since then, the patient was on continuous self-catheterization to aid in the passage of urine. He was on medication to manage constipation though relief was negligible.

**Figure 1:**
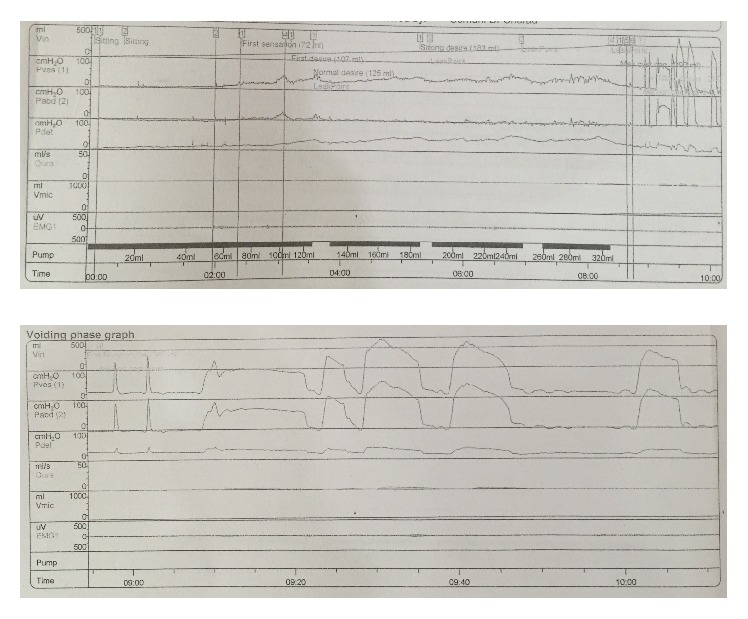
Pressure-flow study report: pretreatment.

Cystometry done prior to cellular therapy revealed reduced detrusor compliance. Voiding sequence also showed absence of voluntary detrusor contraction leading to severe voiding inefficiency and retention. The changes reflected a lesion of the lower sacral nerve roots with an element of fixed distal sphincter obstruction (Figure 1).

### 2.1. Stem Cell Therapy

Cellular therapy with autologous bone marrow mesenchymal and adipose derived stem cells was recommended to the patient. Approximately 100 mL of bone marrow from the right iliac crest and 70–80 mL of adipose tissue from right gluteal region were aspirated under local anesthesia. Mesenchymal stem cells were administered through intrathecal and intramural routes. Under USG guidance, bone marrow and adipose derived mesenchymal stem cells were injected in the transmural, submucosal, and intramural layers of the urinary bladder. The cells were administered at different locations, namely, from the dome to lateral wall and near the neck of the bladder.

## 3. Results

**Figure 2:**
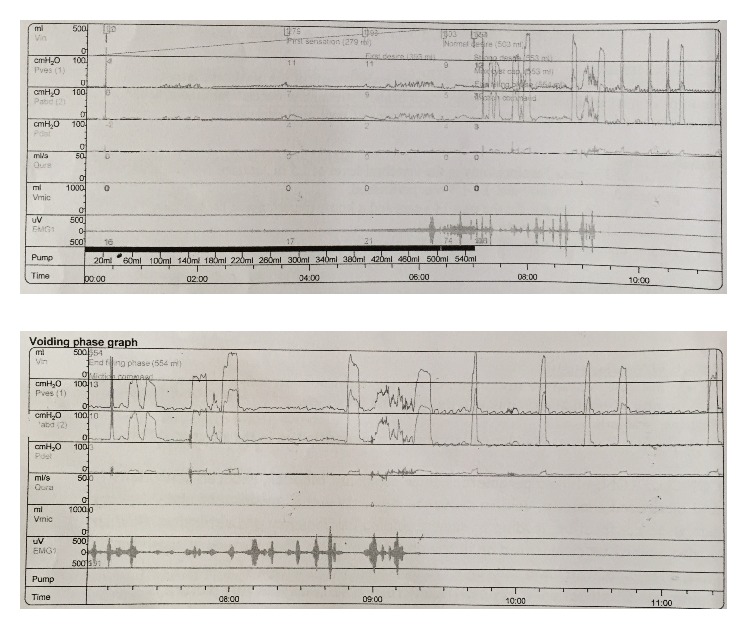
Pressure-flow study report: posttreatment.

The patient was able to micturate voluntarily and catheter was removed on the 10th day following cellular therapy. In the following week, the patient regained some of his ability to defecate voluntarily. Cystometry revealed increased detrusor compliance, reduced sensations, and increased capacity.  Figure 2 shows the pressure-flow study graph done after treatment. One month following cellular therapy, further improvement was noticed in both micturition and defecation. The patient has discontinued the medications for constipation. Pelvic floor strengthening exercises were advised which the patient continues to perform regularly.

## 4. Discussion

Under normal conditions, the detrusor muscle, bladder neck, and striated external sphincter function as a synergistic unit for adequate storage and complete evacuation of urine. The bladder and sphincter are innervated by the spinal conus, which is located at the L1 vertebra. The detrusor muscle is innervated by fibers from 2nd, 3rd, and 4th sacral roots; hence, any lesion in the region or lumbar spinal surgery (as seen in this patient) may lead to neurogenic bladder. In addition, diabetes mellitus may lead to autonomic and peripheral neuropathy and impaired nerve conduction. With long-standing and/or uncontrolled diabetes, decompensation of bladder tissue and function ensues, resulting in hypocontractile detrusor which may lead to urinary voiding problems [2].

Stem cells have shown a good potential for urothelial, neural, and smooth muscle differentiation which are suggestive of the potential uses of cellular therapy in bladder dysfunction [3]. Shukla et al. (2008) reported successful differentiation of stem cells into smooth muscle for bladder repair and replacement [4]. In this case, intramural route of cell transplant was adopted and positive results on bladder function were noticed, which confirms the myogenic differentiation potential of mesenchymal stem cells.

Similarly, the potential of mesenchymal stem cells in spinal cord and other nerve related injuries has been demonstrated. Bone marrow MSCs when transplanted through the cerebrospinal (CSF) fluid have been shown to promote behavioral recovery and tissue repair in spinal cord injury [5]. Hu et al. showed that intravenously transplanted bone marrow stromal cells survived in the L3-4 and had beneficial effects on the recovery of bladder function in the rats after spinal cord injury [6]. In the present study, mesenchymal stem cells were administered by intrathecal route through the CSF, which is a more targeted way of transplantation of cells for spinal cord injury than intravenous route. Axonal regeneration has been shown to be facilitated by intrathecal administration of cells [7].

Adipose derived stem cells (ADSCs) administered through intravenous or transurethral routes have shown significant improvement in increasing elastin content in bladder and voiding function [8]. Also, studies of bladder dysfunction treated with ADSCs have shown high levels of smooth muscle actin and smoothelin expression. These structural proteins have been implicated in regeneration of muscle tissue. Although ADSCs have the capability to differentiate into smooth muscle cells, the main mechanism appears to be paracrine activity which reduces apoptosis and preserves the “suburothelial capillary network” [9].

Chun et al. proposed a triple stem cell therapy approach using human amniotic stem cells. They stated that combined injection of three different lineages of early-differentiating human amniotic fluid-derived cells (myogenic, neurogenic, and endothelial) restores urethral sphincter function [10]. Despite the positive results in the abovementioned study, the inherent disadvantage lies in the source of stem cells. The advantage of using autologous stem cells lies in its safety and ease of harvesting. MSCs can be isolated and expanded to give the needed therapeutic dose.

The mechanism of therapeutic action of stem cells in neuronal injury is most probably a combination of functions: reduction of apoptosis and hence inflammatory response, stimulation of endogenous neurogenesis, induction of angiogenesis, and formation of new neuronal connections in addition to the paracrine action [11].

## 5. Conclusion

Regenerative medicine and cellular therapy is a promising therapeutic approach for organ dysfunction and tissue replacement. The administration of BMSC and ADSC through appropriate routes in this case provided a mixed population of cells and growth factors which enabled us to harness the maximum potential of the cells. Nevertheless, regular follow-up to monitor progression/improvement of the condition is mandatory. Also, further trials and novel routes of administration are necessary to improve efficacy of cellular therapy in various conditions.
